# Reducing sickness absence and stigma due to mental health difficulties: a randomised control treatment trial (RCT) of a low intensity psychological intervention and stigma reduction programme for common mental disorder (Prevail)

**DOI:** 10.1186/s12889-023-16200-x

**Published:** 2023-07-10

**Authors:** Nicola S. Gray, Helen Davies, Rhodri Brad, Robert J. Snowden

**Affiliations:** 1 Department of Psychology, Swansea University, Swansea, Wales SA2 8PP UK; 2 Caswell Clinic, Swansea Bay University Health Board, Bridgend, UK; 3 Driver and Vehicle Licensing Agency (DVLA), Swansea, UK; 4 School of Psychology, Cardiff University, Cardiff, UK

**Keywords:** Randomised Control Trial, Prevail, Work-based intervention, Self-stigma, Stigma, Absenteeism, Low-intensity psychological interventions, Co-production, Absenteeism

## Abstract

**Background:**

Common mental disorders are the leading cause of workplace absences. The Prevail intervention programme aims to reduce stigma and to educate staff and managers about evidence-based low intensity psychological interventions for common mental disorders (depression, anxiety, stress, and distress). Prevail is innovative in taking a public health approach. It is designed to be given to all employees irrespective of their past or current mental health. Prevail was evaluated in three studies examining: (1) the acceptability of the intervention and perceived usefulness; (2) whether the intervention altered stigmatic attitudes and motivation to seek help; and (3) whether the intervention reduced sickness absence, both overall and due to mental health problems.

**Methods:**

A two-armed cluster randomised control trial (RCT) evaluated the effectiveness of Prevail. Employees (*N* = 1051) at a large UK government institution were randomised to an active intervention or control arm in teams identified by their managers (*n* = 67). Employees in the active arm received the Prevail Staff Intervention. The managers in the active arm also received the Prevail Managers Intervention. Participants’ satisfaction and analysis of the Prevail Intervention were gathered by a bespoke questionnaire. Questionnaire measures of attitudes to mental health and mental health stigma were taken 1–2 weeks prior to the intervention and approximately 4 weeks post-intervention. Data relating to sickness absence were gathered via the official records in the time period 3-month post-intervention and for the same period 12 months earlier.

**Results:**

Prevail was evaluated highly favourably by both the staff and their managers. Prevail produced significant reductions in self-stigma and anticipated stigma due to mental health difficulties. Crucially, sickness absence was significantly reduced by the Prevail Intervention.

**Discussion:**

Prevail achieved its goals of producing a palatable and engaging intervention that altered staff’s attitudes and stigmatic beliefs related to mental health and, crucially, produced a strong reduction in work-pace absenteeism. As the Prevail programme is aimed at common mental health problems and was not specialised to this particular workforce, the study provides the evidence-base for a mental health intervention programme that could be used by many organisations across the world.

**Trial Registration:**

ISRCTN12040087. Registered 04/05/2020. 10.1186/ISRCTN12040087. A full protocol for the randomised control trial was published: Gray NS, Davies H, Snowden RJ: Reducing stigma and increasing workplace productivity due to mental health difficulties in a large government organization in the UK: a protocol for a randomised control treatment trial (RCT) of a low intensity psychological intervention and stigma reduction programme for common mental disorder (Prevail). *BMC Public Health* 2020, 20(1):1–9.

## Background

Common mental disorders (CMDs, i.e. anxiety and depression) contribute around 16–17% of the burden of adult disease in the UK [1]. They are also major factors in sickness absence from work [2–5]. This has significant negative outcomes for both the employer and for the economy due to lost productivity. In this paper we report on the results of a randomised control trial for an intervention programme (Prevail) that aims to improve mental wellbeing and reduce sickness absence due to CMDs. Unlike most intervention programmes, Prevail takes a “public health” approach [6] and targets the whole workforce, rather than only intervening with people with active mental health problems. This avoids having to accurately identify people who have current mental health problems, but instead serves to increase overall levels of mental wellbeing in the workforce via a programme of psychoeducation and application of evidence-based low intensity psychological intervention. A protocol for the study was published [7].

### Previous intervention programmes

Most previous work aiming to look at the effects of therapies for CMDs on employment-related variables (e.g., sickness absence, return to work) have looked at standard treatments for CMDs such as cognitive behavioural therapies or medication. While such treatments have the expected (positive) effect on symptom reduction related to mental health problems, they do not have an impact on return to work and only modest effects on sick leave [8]. Perhaps more effective results might be obtained if there were more workplace-based interventions that involved co-operative sickness management plans that include both the person and their employer working together for the benefit of both (termed co-production).

Workplace interventions specifically target the problem as it affects the person’s ability to function in the workplace and involve the active involvement of the employee. However, such a process is likely to be challenging as the employee and employer may have different perspectives and aims [9]. Nevertheless, there is some, although mixed, evidence that work-based interventions can reduce sick leave due to CMDs [10, 11]. For instance, the systematic review of Dewa et al. [10] identified three studies that had examined return to work rates due to workplace interventions and noted significant effects in two of the studies [11, 12] but not in the other [13]. Six studies also looked at the duration of sickness absence, but only one study found effects due to the intervention [12]. It should be noted, however, that these studies looked at interventions targeted at people who were currently suffering from (or had a recent history of) CMDs. There appears to be no studies that have examined interventions that are aimed at the whole workforce irrespective of current or historical CMDs. Our programme (Prevail) is novel, therefore, in taking a “public health” approach [6] to improve the mental wellbeing of all employees within the organization in the hope that this will also translate into reductions in sickness absence. A significant advantage of this approach is that it does not necessitate the accurate identification and “labelling” of staff who currently have CMDs (with all the associated problems in doing so) as the entire workforce benefits from the intervention.

### Prevail

Prevail is a multi-faceted programme aimed at reducing sickness absence and presenteeism due to CMDs. It involves two psychological interventions, both provided via group based intervention programmes. The first (Prevail Staff Intervention) is for all employees within the organization and its aims are to improve knowledge about mental health, including knowledge of best-practice in low intensity psychological inventions and the theoretical premises underpinning such interventions. It also aims to reduce stigma related to mental health issues, and in particular self-stigma [14], and thus promote help-seeking behaviours both within and outside of the workplace. It covers: (1) the basics of mental health literacy; (2) the normalization of CMDs; (3) attempts to reduce stigma associated with CMDs, with an emphasis on self-stigma (feelings of poor self-worth due to their mental health problems: [15]); and 4) a plan of managing CMDs within the workplace to reduce distress and work-place functional impairment. This includes situations when simple adjustments in work-based practice may greatly assist, when low intensity psychological interventions are appropriate, and when professional psychiatric or medical help may be required.

The second intervention (Prevail Managers Intervention) is aimed at the managerial level within the workplace and is designed to teach managers a formulation-based approach to evaluation and intervention. Formulation refers to a process of providing an explanation for the presenting problem and differs from a “diagnosis” which is more categorical and refers to identification and labelling of the actual CMD rather than an understanding of the causes, or trigger-factors, of the CMD for the individual. The focus here is on the understanding of the problem for the person, active problem-solving, and co-production (where both the employer and the employee share the responsibility to plan and deliver the intervention within the work-place and both make a contribution and commitment to this plan [16]). The aim is to improve mental wellbeing, reduce sickness absence, and enhance productivity.

Prevail was therefore devised as an intervention to improve the mental wellbeing of all employees via education in mental health literacy (including behaviours that improve mental wellbeing), low-level psychological interventions for less severe problems, and encouragement of help-seeking behaviours by destigmatising mental health issues. As such it was not specifically designed for this particular work setting (e.g., DVLA) but as a general programme of prevention/intervention to improve mental wellbeing, predicated upon established evidence-based practice, that could be used in many employment settings. However, in delivering the programme in this employment setting, we used specific examples related to the employer (using videos and case studies of their staff and work environment) so as to emphasise and illustrate the learning points and relevance to the specific workforce.

### Aims and hypotheses

Our original aims were to: (1) measure mental health literacy (and mental health stigma in particular) before and after the intervention, (2) compare sick leave in the 12 months pre- and post-intervention, and (3) to measure quality of life, presenteeism, and current mental health 12 months post intervention [7]. To do this we used a clustered randomised control trial (RCT) in which some managers and those employees under their management were provided with the Prevail intervention while other managers (and those employees under their management) were assigned to a control (no intervention) group. However, due to the onset of the COVID-19 pandemic in the UK (commencing March 2020) there were dramatic changes in the workplace environment, with the vast majority of employees having to work from home. Hence, we had to adapt our overall aims and protocol to adjust for this unprecedented situation. The planned 12-month follow-up after the Prevail Intervention was reduced to 3 months after the delivery of the Prevail Intervention (December 2019- February 2020) and before the onset of COVID restrictions, with the pre-Prevail comparison period being the same 3-month period one year earlier (December 2018- February 2019) so as to avoid possible seasonal effects. We were unable to take the planned 12-month follow-up data on quality of life, presenteeism, and current mental health. We also report on people’s perceptions of the Prevail Intervention which was not covered in our original research protocol.

## Methods

The setting was the Driver and Vehicle Licencing Agency (DVLA). The DVLA is the executive agency part of the Department for Transport. DVLA maintain the registration and licensing of drivers and vehicles in Great Britain. It employs around 6,000 people mainly at its head office in Swansea, Wales, UK. All participants in this study were employed at the Head office.

### Participants

Sixty-seven managers across four divisions of the DVLA, stratified by division [Information Technology Services (ITS), the Contact Centre (CC), Casework and Enforcement Group (CAEG), and Input Services Group (ISG)] were chosen for the study. These were then randomised (using a random number sequence) into the two arms (active vs. control) with stratification to ensure equal numbers of managers from each division and similar gender profile being assigned to each group. These procedures were implemented by staff within the Human Resources department of the DVLA. The “random” split did not achieve a perfectly even split and 59% of the staff were allocated to the active arm of the study.

**Fig. 1:**
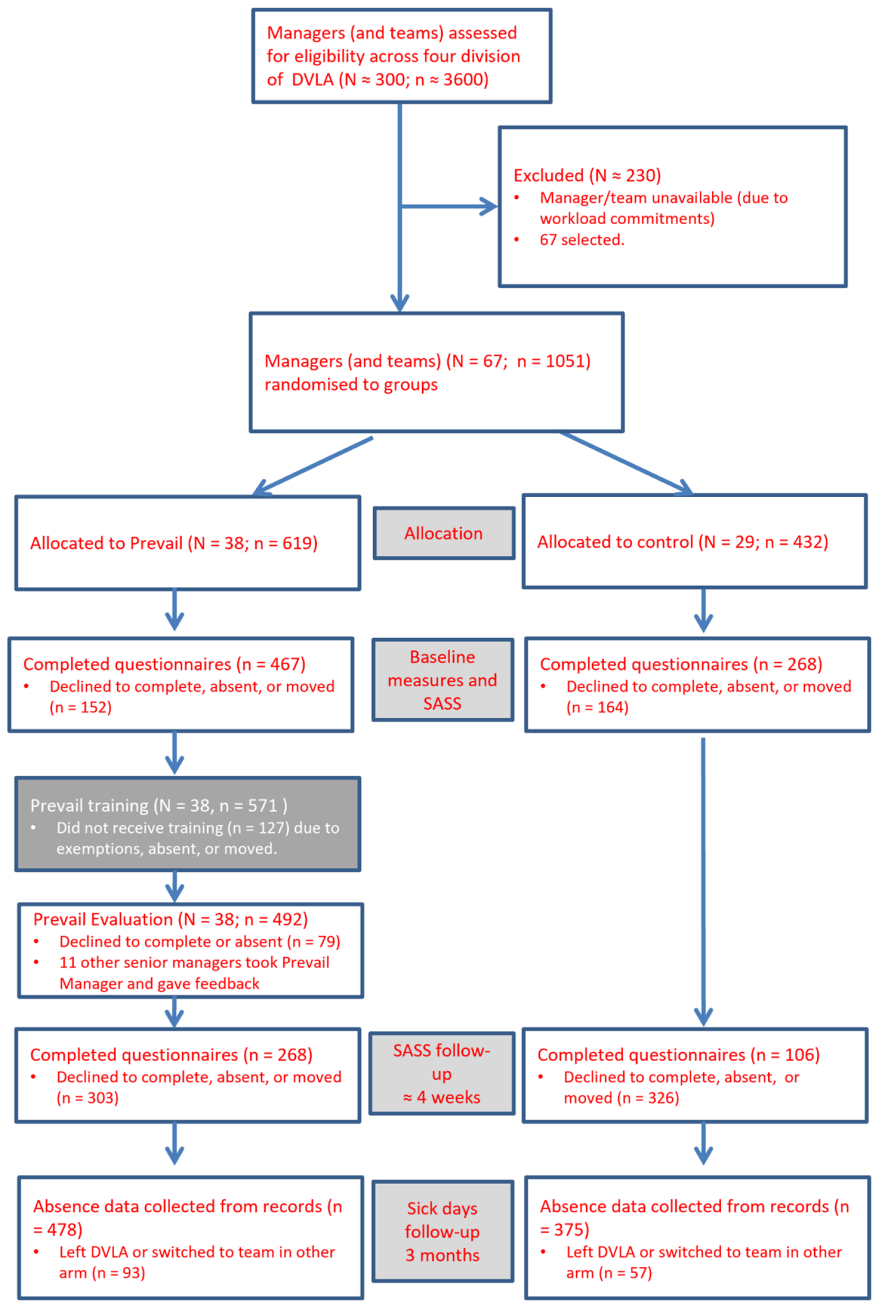
Consort Representation of Study

The numbers in each phase of the study varied due to issues such as staff moving from the DVLA during the study, staff moving between teams/divisions within the DVLA, or not agreeing to consent to the study, etc. Numbers at each stage are illustrated in Fig. 1.

### Power and statistical analysis

Our initial aim (see [7]) was to recruit at least 46 managers and therefore 552 employees (as each manager was estimated to manage an average of 12 people) based in a power analysis of an alpha = 0.05, power of 80%, a standardised effect size of 0.30, and design effect due to the clustered nature of the RCT of 1.55 (for details of this see [7]). However, we deliberately over-recruited participants as we expected a proportion of the sample to move between managers over the period of the study, and that some would not give consent to complete the questionnaire parts of the study. However, due to limitations in what data were available to us due to the UK Data Protection Act (2018) and the Data Protection Impact Assessment (DPIA) completed by the DVLA, we were not able to obtain information relating to which specific manager each participant belonged to and hence we could not use a clustered design for the statistical analysis.

A *post-hoc* sample size calculation was performed to evaluate adequate statistical power to detect any differences between the active and control arms for the sickness absence data. Using a two-tailed hypothesis, a hypothesized difference of 14% between the two treatment arms, an alpha value of 0.05, a beta value of 0.20, and equal allocation to treatment arms, a total of *n* = 372 participants, with *n* = 186 in each treatment arm would be needed to have enough statistical power in the study. The sample size calculation was performed using G*Power Version 3.1. Hence, both our total sample size with complete data for the sickness absences (N = 853), and number of participants in the individual arms (Active = 478; Control = 375), were well above these requirements.

### Prevail intervention

The Prevail intervention programmes were conceptualised and written by two of the authors (NSG, RJS) who also served to train the trainers that delivered the Prevail Staff Intervention and to deliver the Prevail Managers intervention. NSG is a Consultant Clinical and Forensic Psychologist with over 25 years of experience working with people with mental health problems within both the NHS and with independent healthcare providers. She is a Professor of Psychology at Swansea University teaching courses to masters students on Clinical Psychology and mental health. She has over 100 peer reviewed publications in this area of research. RJS is a Professor of Psychology at Cardiff University with over 25 years of teaching and research in diverse areas of psychology, including mental health.

The Prevail intervention consists of two parts. Part one, the Prevail Staff Intervention, is targeted at all staff. This involves attendance at a one-day intervention programme that incorporates a number of psychological techniques designed to: (1) improve knowledge about mental health and cover the basics of mental health literacy; (2) enhance the normalization of common mental disorder; (3) reduce stigma associated with common mental disorder, with an emphasis on self-stigma; and (4) assist staff to learn how to formulate a plan of managing common mental disorder within the workplace to reduce distress and workplace functional impairment. The Prevail Staff Intervention includes information about evidence-based low intensity psychological interventions for common mental health disorders. This includes intervention strategies for depression, anxiety, stress, and distress (including bereavement). The intervention also actively encourages disclosure of mental health difficulties and appropriate help seeking behaviour.

Part two, The Prevail Managers Intervention, teaches managers the skills of active problem-solving interventions, formulation-based approaches to intervention, and co-production of solution-focussed management in order to support and intervene with staff currently experiencing, or at risk of developing, a CMD. The philosophy behind this managerial intervention is that mental health difficulties do not occur in a social vacuum and that if staff and their managers can be taught evidence-based active problem-solving interventions and the methodology of co-production [17], this should greatly enhance their ability to remain in the workplace and be resilient to negative outcomes of poor mental health. Consistent with this, Gilbreath and Benson [18] found that line managers play a crucial role in employees’ quality of experience in the workplace and that the behaviour of managers predicted the outcome of mental health and psychiatric disorder over and above variables such as age of employee and level of social support at home. They concluded that supervisor and managerial behaviour is an important determinant of employees’ psychological well-being and should not be neglected in psychological interventions and research that attempts to improve work-place mental health.

### Delivery of prevail

The Prevail Intervention programme consists of a series of nine modules taking a mainly didactic approach to learning, but with many interactive components and case study discussions to enhance participant engagement. The modules consist of lectures on specific topics (e.g. Module 5 “Stress and Emotional Stress”) that introduce the topic, give examples of these problems, and provides psychoeducation about the evidence-based approaches to reduce the problem or deal with more serious difficulties in this area. The lecture materials are complemented with video case study examples (produced within the DVLA in this instance), group activities, group discussions, and revision quiz(s). The Prevail Manager’s Intervention uses a similar approach.

A Train the Trainer approach [19] was taken in which six employees of the DVLA were selected to deliver the Prevail Staff Intervention. The Prevail trainers were selected by the DVLA to be effective trainers with good social skills, but also to have a positive attitude and interest in mental health and well-being and a good baseline knowledge about mental health difficulties and CMDs. These people were then trained by the authors (NSG, RJS) on both the content of Prevail Staff Intervention and on teaching techniques. These trainers were given time from their normal duties to learn the Prevail Intervention and practiced with each other and with members of the Human Resources team on effective programme delivery. The Prevail trainers then delivered the Prevail intervention programme to employees in the intervention arm of the RCT. Prevail was delivered via one-day in-person workshops to groups of 10–20 employees.

Delivery of the Prevail Staff Intervention to those in the active group commenced on the 30th October 2019. Fifty-seven staff cohorts received the Prevail programme via group-based intervention (N = 571). The cohorts consisted of staff teams with other colleagues working in the same division, and where possible with the same manager. Where staff were unable to attend the allocated day of Prevail delivery they were individually re-scheduled to attend another session. Some staff teams were split over two days to accommodate working patterns.

The Prevail Managers Intervention programme was delivered by the authors of Prevail (NSG, RJS) jointly with the Head of Talent and Learning at DVLA over three sessions to the 38 managers that had been allocated to the intervention arm of the study (commencing October 2019). It was important that there was a close association between the delivery of the Prevail Staff Intervention and the Prevail Managers Intervention as, if our intervention was successful in its aims of reducing stigma and enhancing help-seeking, the managers had to be ready and skilled to address the issues of staff as they arose. Eleven other senior managers also attended one of these sessions in order to become familiar with the intervention and aims of the Prevail programme.

### Measures

***Opinions on the Prevail Programme.*** On completion of the Prevail Staff Intervention programme participants were given a feedback sheet to complete (via an anonymised electronic survey). Questions were answered on a 5-point Likert scale of strongly agree, agree, neither agree or disagree, disagree, or strongly disagree. For simplicity, we have rescored these categories into agree, neutral, or disagree by combining the top two and bottom two categories. Participants were also asked open-ended questions about why they agreed or disagreed with a particular question. A similar measure was administered to participants after the Prevail Managers Intervention.

***Mental Health Literacy and Stigma***. The Stigma and Self Stigma scales (SASS; [20]) is a 42-item questionnaire that measures attitudes towards mental health problems and includes the following subscales: stigma to others, social distance, anticipated stigma, self-stigma, avoidant coping, and (lack of) disclosure/help-seeking. The SASS also contains items related to social desirability (otherwise termed positive impression management) that are not related to mental health issues in order to identify those individuals who are engaging in response bias and are giving an overly positive view of themselves [21]. Participants respond to each statement using a five-point Likert scale (strongly agree, agree, neither agree nor disagree, disagree, strongly disagree), which is scored 0 to 4. Some questions were reverse scored to prevent response bias. Each of the seven sub-scales (including social desirability) had six items and sub-scale scores could range from 0 to 24. Previous research [20] developed and measured the psychometric properties of the SASS. This research found that all scales of the SASS have acceptable test-retest reliability over a 4-week period (*r*s > 0.67) and good internal reliability (*r*s > 0.62). However, there was one exception to this in that the Avoidant Coping scale did not reach acceptable levels of internal consistency (*r* = .42) and so this scale was not analysed further in this report.

***Sickness Absence Data.*** Data from Human Resources (HR) records for the staff and managers in the study were processed by the DVLA Human Resources staff. This data was communicated to the researchers only at a group level (e.g., average number of sick days in the active group as compared to the control group over the 3-month period post intervention and for the same 3 month period one year earlier (pre-intervention)), separated by gender and age, etc. This was in order to ensure that the sickness absence data was anonymous to the research team and to comply with the UK Data Protection Act (2018) and Data Protection Impact Assessment (DPIA). These analysis periods differed from our original protocol [7] due to the onset of the COVID-19 pandemic and concomitant changes to work practices.

### Design

A clustered randomised control trial (RCT) design was deemed necessary as the Prevail programme addresses the two-way communication of mental health education and information, planning of active problem-solving and use of low intensity evidence-based mental health interventions (and/or help seeking) between a manager and the employees within their team. Hence, both the manager and all members of their team have to be in the same arm of the intervention and the only way to achieve this was via a clustered RCT design. Hence, randomisation took place at the level of the managers. Each manager manages approximately 12 people (although this varied from division to division within the DVLA).

***Wave 1.*** The first week of data collection took place 1–2 weeks prior to the Prevail intervention for the active group, with data collection for the control group being yoked to this (but with participants and researchers being blind to which group each cohort of participants were in). This provided baseline measures of levels for the SASS, and information about current mental health and well-being.

***Wave 2.*** This occurred approximately 4 weeks after the participant had engaged in the Prevail intervention for the active group, with data collection for the control group being yoked to this. Its aim was to examine if the Prevail intervention was able to change attitudes about mental health, reduce stigma, and improve help-seeking behaviour.

***Wave 3.*** This was originally designed to be 12 months after the Prevail intervention for the active group, with data collection for the control group being yoked to this [7]. However, due to the COVID-19 pandemic we were not able to take these measures.

It should be noted that the managers and participants in the active and control arms of the study worked “side by side” and it is very likely that there would be “leakage” and contamination of information in the Prevail intervention programme from the people in the active arm to those in the control arm (and we have anecdotal reports of such instances). While this might be seen as a “positive” in that the Prevail intervention might have effects outside of those who actually received the programme, it would have had detrimental effects on our RCT as the “control” participants may have also indirectly benefitting from the Prevail programme. The issue of treatment contamination is perceived to be a significant problem in trials using complex interventions for mental health issues, such as in the present study [22]. Magill et al. [22] suggest that the most common design approach to deal with contamination is the clustered randomisation technique which we used in the present study. We note that such leakage would serve to reduce our ability to show differences between the active and control participants due to the Prevail intervention.

## Results

### Part 1. Perceptions of the prevail training

**Fig. 2:**
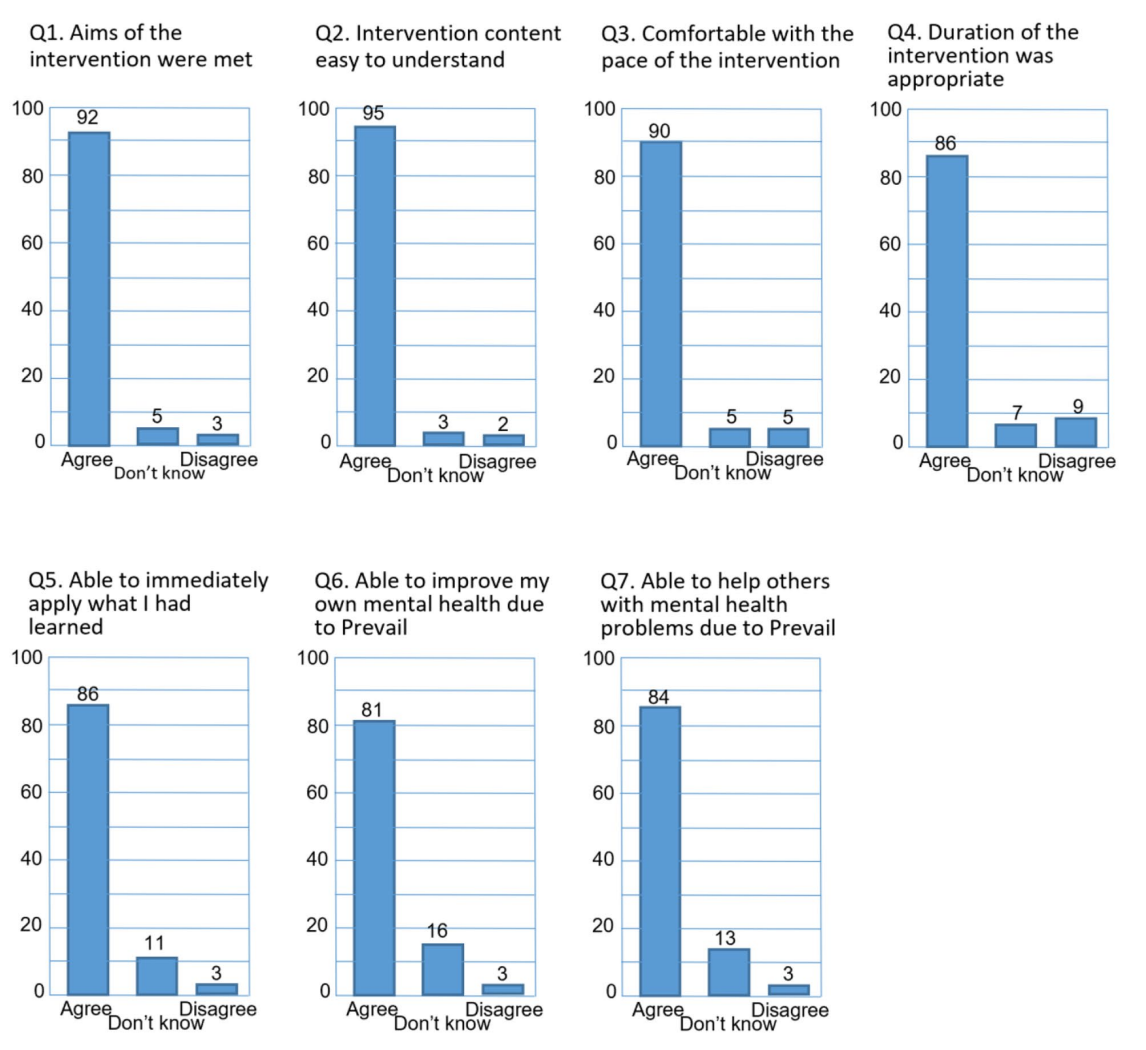
Quantitative Feedback from Prevail Staff Intervention

Overall, 492 people completed the evaluation of the Prevail intervention. No demographic information was taken. A summary of the quantitative results for the Prevail Staff Intervention is given in Fig. 2. The four questions relating to the palatability of the Prevail Staff Intervention all received overwhelming endorsement: 92% of participants thought the aims of the intervention were met, with 95% finding the content easy to understand. For those that disagreed that the intervention was easy to understand, the comments mainly focused on physical reasons, in particular the sound quality of the videos. 90% of participants thought the pace of the intervention was appropriate and 86% felt its duration was appropriate. Those that disagreed generally wanted a faster and shorter intervention. Hence, overall, we can conclude that the majority of participants were happy with the intervention programme.

Importantly, three questions in the quantitative evaluation aimed to examine if participants felt the intervention had equipped them with the knowledge and skills that had been intended: to be able to apply in practice what they had learnt; to be able to improve their own mental health; and to be able to help and assist others with their mental health difficulties. Again, these questions elicited very positive responses. 92% of participants endorsed that the aims of the intervention were met. 86% of people felt that they were able to immediately apply what they had learnt about mental health and evidence-based low intensity psychological interventions, and 81% of people felt that they were able to help others with their mental health difficulties (compared to 3% disagreeing for each of these latter two questions). Examination of the responses of those who did disagree identified the theme of “*there was nothing new/I already do this*” which was identified by seven respondents. There will clearly be some people in any organisation who have a history of mental health difficulties and who have had the benefit of already receiving low intensity psychological intervention or cognitive behavioural therapy. For these people, a lot of the content of Prevail may not be new (as Prevail has its foundation in evidence-based psychological practice) and may be more of a revision and reinforcement of effective interventions for mental health and well-being. Hence, overall, we can conclude that the majority of participants felt the intervention programme had achieved the core aims of using positive strategies to improve their own mental health and to support the mental health difficulties of others.

Thematic examination of responses to the question “What (if any) part(s) of the course did you find useful?” revealed four major themes. The most common theme (56 respondents) was termed “all of it” which is exemplified by the comments:

- “*Everything about the course. I really enjoyed it and have suffered with mental illness myself”;* and.
- “*I feel overall it will create a great attitude in the workplace around mental health*”.

The theme “videos and case studies” was reported as being particularly useful by 50 respondents, where they expressed that seeing real people (and their colleagues in particular) talking about their mental health difficulties and well-being, enhanced their understanding of these issues (with one video and case study included for each of anxiety, depression, stress, and bereavement).

For example:

- “*I found the real-life interviews with colleagues very beneficial, to see that these people are all around us and part of our lives was eye opening*.”.

The theme “mental health as a continuum” was mentioned as being particularly useful by 22 respondents and as important in tackling stigma and self-stigma.

For example:

- “*The mental health continuum was particularly useful and that everyone is in the same boat*”.

Finally, 16 people mentioned the section on “stress” as being particularly useful, with the theme emerging that this is often ignored and not treated as a real or significant problem impacting mental health and well-being in the workplace.

For example:

- “*I particularly found the section about stress the most useful as it’s something that applies to everyone. Whereas depression and anxiety is something that’s regularly talked about and advertised everywhere, stress doesn’t usually get talked about in as much detail, which is why I found it the most interesting*”.

**Fig. 3:**
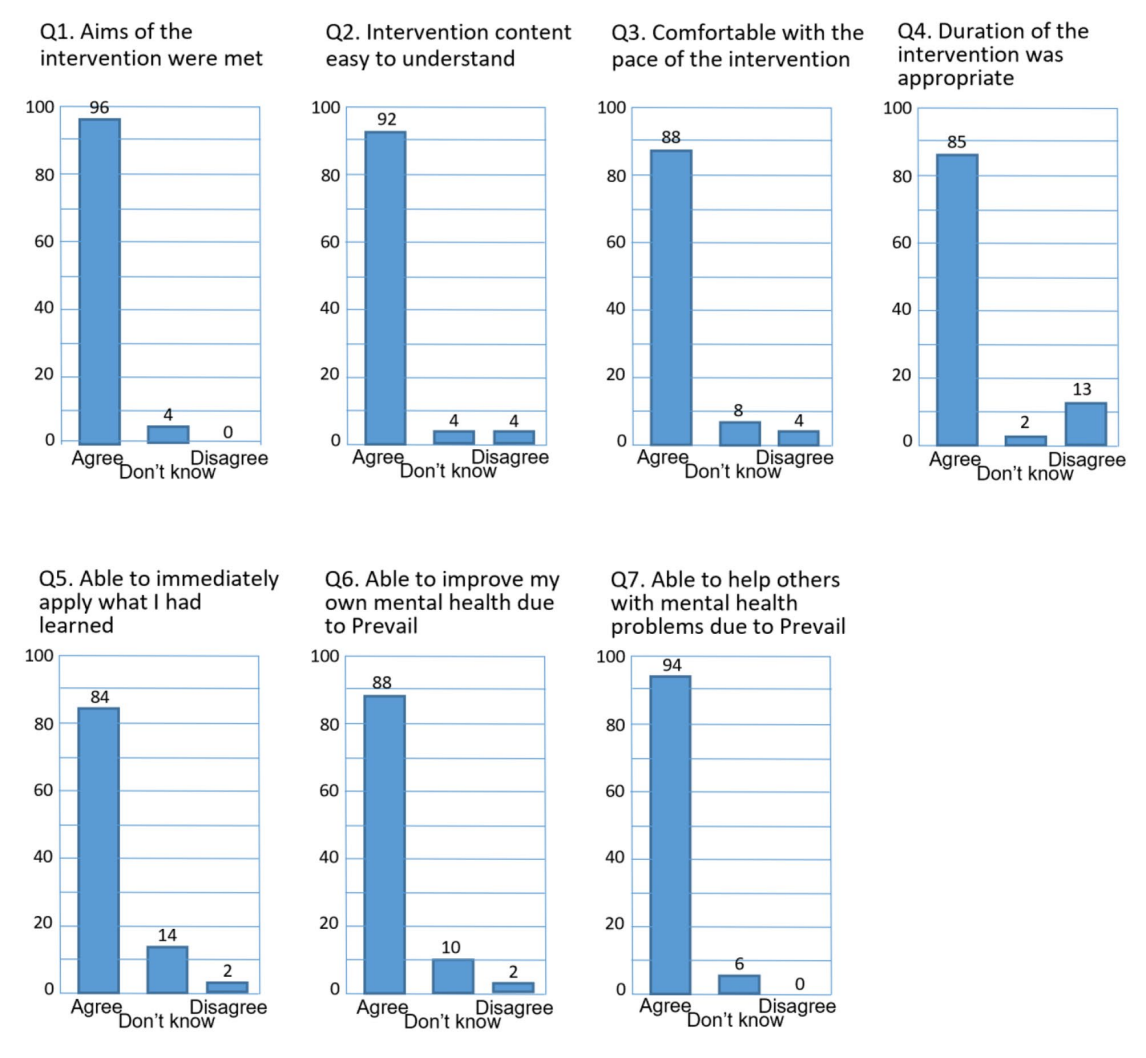
Quantitative Feedback from Prevail Managers Intervention

In total 49 people undertook the Prevail Managers training. This included the 38 managers in the active arm of the study alongside 11 other senior managers not directly involved in the main study. No demographic information was taken. A summary of the quantitative results for the Prevail Manager Intervention is given in Fig. 3. The four questions relating to the palatability of the Prevail Manager Intervention programme all received overwhelming endorsement: 96% of managers thought that the aims of the intervention were met, 92% finding the content easy to understand, 88% of managers thought that the pace of the intervention was appropriate, and 85% felt its duration was suitable. Examination of those that disagreed with any of these statements did not establish any common comments or themes, with the reasons given being contradictory (e.g. one person thinking the intervention programme was “too slow” whereas another felt it was “too fast”).

Three questions aimed to examine if managers felt that the intervention programme had equipped them with the knowledge and skills that had been intended: to be able to apply in practice what they had learnt; to be able to improve their own mental health; and to be able to help and assist others with their mental health difficulties. Again, these questions elicited positive responses. Particularly encouraging were the responses to the question about being able to help others, as this was integral to the aims of the Prevail Managers programme. 94% of managers agreed that Prevail Managers had improved their ability to help others with their mental health difficulties, compared to 0% who disagreed. Examination of the responses of those who were neutral on this question (neither agreeing nor disagreeing) showed very few comments with no common theme.

Thematic examination of responses to the question “What (if any) part(s) of the intervention did you find useful?” revealed one theme mentioned by eight respondents. This consisted of comments stating that they found the section of the intervention programme related to *“Active Problem Solving”* to be particularly beneficial, but managers did not provide more detail than that.

The feedback for both the Prevail Staff Intervention and the Prevail Manager Intervention programmes has shown an overwhelming endorsement by staff and managers that the aims of the intervention programme have been met, the content of the intervention is fit for purpose, and the delivery of the programme is satisfactory.

We thought it useful to summarise with two comments from delegates:

- “*The whole course was very informative and useful. It has encouraged myself to improve my mental health. It also allows me to help colleagues, friends and family.”*
- *“I hoped to enjoy the course prior to joining and must say that I did. Interesting topics, good conversations, thought invoking case studies and a good pace all helped. Plus, the phrase ‘a sexy herd of zebras’ has never been said at any other course I’ve attended and was a highlight! Thank you.”*

### Part two. Changes in mental health literacy

The descriptive statistics for the sample, stratified by condition (active vs. control) and wave (pre-assessment or Wave 1 vs. post-assessment or Wave 2) are presented in Table 1. Chi-square tests did not reveal any significant differences (all *p*s > 0.05) between the groups on any of these variables. Unfortunately, the number of completed questionnaires was considerably below our expected levels, particularly for the control group in the second wave of data collection. This is likely to be because these staff and managers were not receiving the Prevail Intervention programme and may not have felt the need to complete the questionnaires on a repeat occasion.

**Table 1:** Demographic Information on RCT participants

		Active Wave 1	Control Wave 1	Active Wave 2	Control Wave 2
Number		467	268	200	106
Female (%)		303 (64.6)	153 (55.6)	140 (70.0)	72 (67.9)
Age (%)	18–29	68 (14.5)	57 (20.7)	26 (13.0)	31 (29.3)
	30–39	135 (28.8)	90 (32.7)	65 (32.5)	40 (37.7)
	40–49	108 (23.0)	53 (19.3)	42 (21.0)	16 (15.1)
	50–59	121 (25.8)	51 (18.5)	52 (26.0)	16 (15.1)
	60+	33 (7.0)	17 (6.2)	15 (7.5)	2 (1.9)
History of MI (%)		229 (48.8)	111 (40.4)	78 (39.0)	44 (41.5)

The psychometric properties for the SASS scales were highly similar to a previous report of the properties of SASS in a workplace population [20]. This included the poor reliability of the Avoidance Scale, which was therefore omitted from further analysis.

### Effects of prevail on attitudes: stigma and self-stigma scale (SASS)

To examine if the Prevail programme had significant effects upon mental health attitudes each of the scales of the SASS was examined in turn using univariate two by two analysis of variance (ANOVA), with factors of intervention condition (active vs. control) and wave (pre- vs. post-intervention). Prior to statistical analysis data were inspected for suitability for ANOVA and all were deemed to be acceptable.

**Fig. 4:**
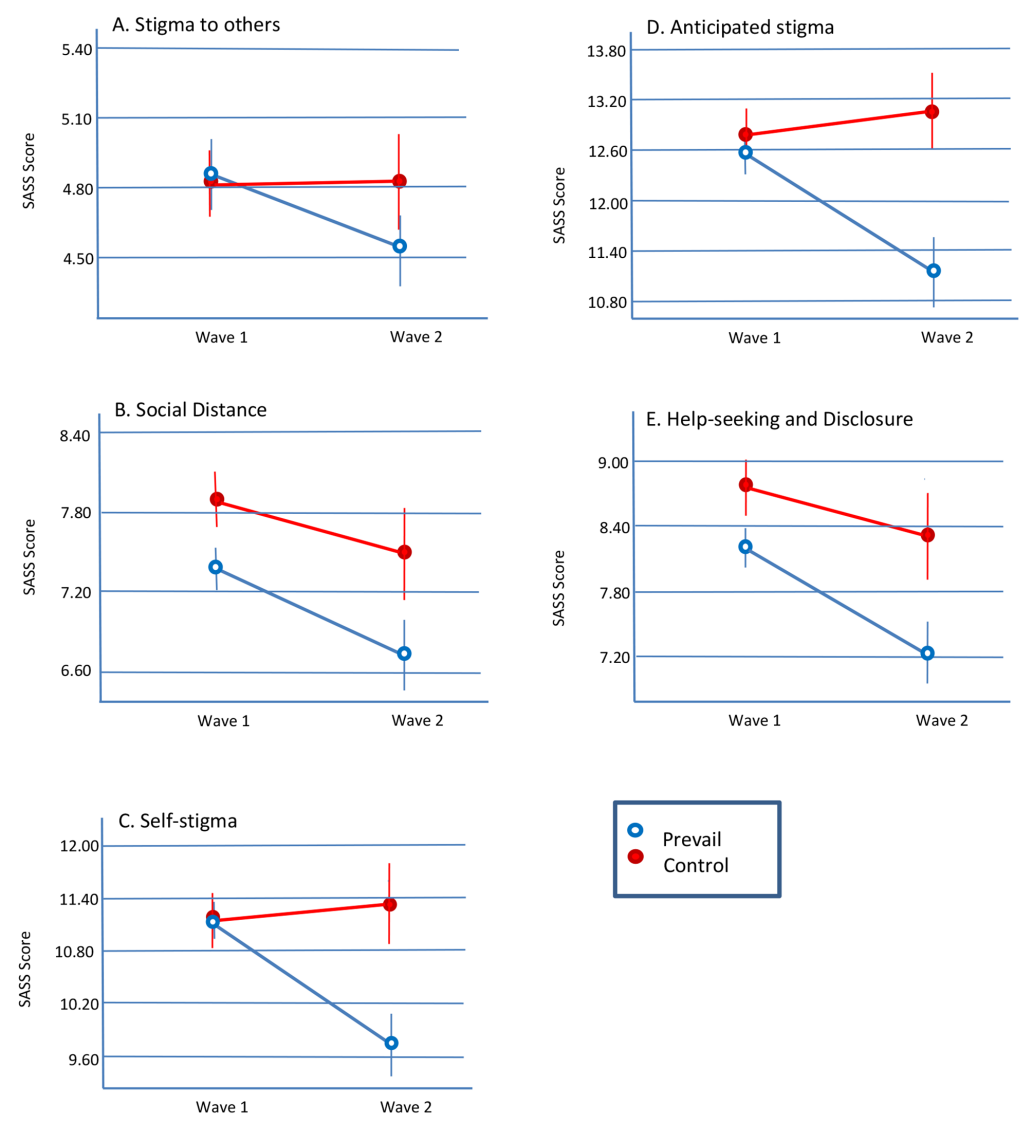
Effects of Prevail on SASS scores. Error bars represent ± SEM.

***Stigma to others.*** Stigma to others refers to a person’s negative beliefs about people with mental disorders (e.g. “*People with mental disorders are weak*”). The results are depicted in Fig. 4a which illustrates that there may be a modest effect of Prevail in reducing levels of stigma to others, but no effect in the control group. However, the main effects of intervention condition (*F*(1, 1020) = 0.28, *p* = .60) and wave (*F*(1, 1020) = 0.39, *p* = .52) were not significant, and neither was the intervention by wave interaction (*F*(1, 1020) = 0.42, *p* = .52). Hence, there was no evidence that Prevail had produced any change in people’s stigmatic attitudes about mental health to other people. However, it may be noteworthy that scores on this scale were very low in the pre-intervention (Wave 1) stage. Thus, the small reduction in stigmatic attitudes after Prevail which is apparent in Fig. 4 may have been subject to a floor effect (i.e., this was so low already it was not possible to bring it down still further; see Discussion).

***Social Distance.*** Social distance is another form of stigma to others, but is more related to the “affective”, or emotional, component of stigma and how close people want to be to a person with a mental health problem (e.g. “*If I were an employer, I would feel comfortable employing someone with a mental disorder*” (reverse scored)). The Social Distance scale measures acceptance of people with mental health problems into their communities (be that the work-place, family/friends, or their local neighbourhood). The results are depicted in Fig. 4b which illustrates that the Prevail intervention reduced levels of Social Distance for the Prevail group (and, therefore, increased acceptance of people with mental health difficulties). However, surprisingly there also appears to have been a decrease in Social Distance in the control group. This is supported by the statistical analysis which found that there were main effects of intervention condition (*F*(1, 1022) = 6.49, *p* = .01) and of wave (*F*(1, 1022) = 4.33, *p* = .04). However, there was no significant intervention by wave interaction (*F*(1, 1022) = 0.25, *p* = .62). An *a priori* t-test showed that participants’ acceptance of people with mental health difficulties was increased by the Prevail programme (*t*(654) = 2.23, *p* = .03). This improvement over time did not reach significance for the control group (*t*(368) = 0.93, *p* = .35). Thus, Prevail was successful in increasing acceptance of (or decreasing social distance from) people with mental health problems.

***Self-stigma.*** Self-stigma refers to what a person thinks about themselves if they have, or were to have, a mental health problem (e.g. “*If I had a mental disorder, I would feel ashamed*”). The results are depicted in Fig. 4c which illustrates that the Prevail intervention has reduced levels of Self-stigma for the Prevail group. This is supported by the statistical analysis which found that the main effect of intervention condition (*F*(1, 1022) = 5.25, *p* = .02) was significant while that of wave (*F*(1, 1022) = 3.15, *p* = .07) was marginally significant. As predicted, there was a significant intervention by wave interaction (*F*(1, 1022) = 5.13, *p* = .02). An *a priori* t-test showed that participants’ Self-stigma was reduced in the Prevail programme (*t*(654) = 3.23, *p* < .001) while there was no such reduction over time for the control group. Hence, the Prevail intervention was successful in reducing levels of self-stigma.

***Anticipated stigma.*** Anticipated stigma refers to what people think other people would think about them if they have, or were to have, a mental health problem (e.g. “*If I had a mental disorder, I would worry other people would think of me as a failure*”). Anticipated Stigma is believed to be strongly associated with Self-stigma in that if a person believes negative things about themselves due to their mental health difficulty, they will also be highly likely to believe that others hold the same negative views of them. The results are depicted in Fig. 4d which illustrates that the Prevail intervention has reduced levels of Anticipated Stigma for the Prevail group. This is supported by the statistical analysis which found that the main effect of intervention condition was significant (*F*(1, 1016) = 8.84, *p* = .003) while that of wave (*F*(1, 1016) = 2.56, *p* = .11) was not significant. As predicted, there was a significant intervention by wave interaction (*F*(1, 1016) = 5.54, *p* = .02). An *a priori* t-test showed that participants’ Anticipated Stigma was reduced by the Prevail programme (*t*(650) = 3.03, *p* < .001) while there was no such reduction over time for the control group. Hence, the Prevail intervention was successful in reducing levels of Anticipated Stigma.

***(Lack of) Disclosure/Help-seeking.*** The (lack of) Disclosure/help-seeking scale examines the reluctance of an individual to disclose or seek help for a mental health problem (e.g. “*I would not feel comfortable discussing my mental health problems with a colleague*”). The results are depicted in Fig. 4e which illustrates that the Prevail intervention decreased levels of Lack of help-seeking (i.e., increased disclosure about mental health difficulties and increased help-seeking behaviour for mental health difficulties). However, interestingly there also appears to have been an increase in help-seeking and disclosure in the control group. This is supported by the statistical analysis which found that there were main effects of intervention condition (*F*(1, 1020) = 7.66, *p* = .006) and of wave (*F*(1, 1020) = 5.73, *p* = .02). However, there was no significant intervention by wave interaction (*F*(1, 1020) = 0.76, *p* = .38). An *a priori* t-test showed that participants’ lack of help-seeking/disclosure was decreased by the Prevail programme (*t*(652) = 2.75, *p* = .006). This improvement in help-seeking and disclosure over time did not reach significance for the control group (*t*(368) = 0.93, *p* = .35). Thus, the Prevail intervention programme was successful in increasing help-seeking and disclosure.

The results show clear effects of the Prevail Intervention programme on levels of self-stigma and anticipated stigma. There were also significant reductions in levels of social distance, and (lack of) disclosure/help-seeking behaviours for those in the intervention arm of the study. However, there were also reductions in these scale scores for those in the control arm of the study. This latter effect was somewhat surprising. We suspect that these reductions in scores in the control participants were due to “leakage” of the Prevail Intervention to people in the control group. The implementation of Prevail within the organisation may have raised awareness of mental health issues in general, and it seems inevitable (and, perhaps, desirable) that people in the intervention arm of the study would have discussed the content and aims of the intervention with their colleagues, including those in the control arm. Hence, the impact of the Prevail Intervention was probably not strictly confined to those in the active arm of the study.

### Part three. Sickness absence

#### Statistical methods

Chi-square analysis was performed to compare the active and control arms on demographic characteristics including age, gender, and directorate. Chi-square analysis and unadjusted odds ratios (OR) with 95% confidence intervals (95% CI) were used to compare the treatment arms on the number of “all sickness” and “mental disorder” days taken as sick leave between two time periods. Finally, Cochran-Mantel-Haenszel tests were employed to compare men and women on their respective “all sickness” and “mental disorder” days taken as sick leave from work. Frequency and percentage statistics were reported for each of the analyses in a cross-tabulation format. Statistical significance was assumed at an alpha value of 0.05 and all analyses were performed using SPSS Version 29 (Armonk, NY: IBM Corp.).

#### Statistical results

The results of the demographic variable comparisons are presented in Table 2. There were no differences between the active and control arms for age, *p* = .59, or gender, *p* = .25.

**Table 2:** Demographics and chi-square analysis for the participants in the RCT for sickness absence

Variable/Level	Prevail	Control	*p*-value
Age			
16–29	83 (17.4%)	66 (17.6%)	
30–49	237 (49.6%)	197 (52.5%)	
50+	158 (33.1%)	112 (29.9%)	0.59
Gender			
Female	345 (72.2%)	257 (68.5%)	
Male	133 (27.8%)	118 (31.5%)	0.25

For the analysis of summary sickness absences, there was a significantly higher proportion of “all sickness” absence days in the control group during the second period of observation (*n* = 1147, 58.4%) versus the active group (*n* = 817, 41.6%), *Χ*^*2*^(1) = 99.33, *p* < .001, OR 1.79, 95% CI 1.60–2.01. For “mental disorder” absences, there was also a significantly higher proportion of control group absent days (*n* = 311, 68.1%) versus the active group (*n* = 146, 31.9%), *Χ*^*2*^(1) = 79.06, *p* < .001, OR = 2.74, 95% CI 2.19–3.44. See Table 3 for the frequency statistics related to summary absences.

**Table 3:** Analysis of Summary Absences (days taken as sick leave)

Variable/Level	Prevail	Control	*p*-value
All Sickness			
Pre intervention	1684 (56.1%)	817 (41.6%)	
Post intervention	1320 (43.9%)	1147 (58.4%)	< 0.001
Mental Disorder			
Pre intervention	701 (56.3%)	146 (31.9%)	
Post intervention	545 (43.7%)	311 (68.1%)	< 0.001

For the comparison of gender related to absences, significant conditional independence was detected between females and males for “all absences,” *Χ*^*2*^(1) = 99.73, *p* < .001. Conditional independence was also detected between males and females for “mental health absences,” *Χ*^*2*^(1) = 131.80, *p* < .001. See Table 4 for the frequencies and percentages related to the Cochran-Mantel-Haenszel findings.

**Table 4:** Gender Analyses of Absences (days taken as sick leave)

Analysis	Condition	Group	Pre intervention	Post intervention	*p*-value
All absences					
	Female				
		Prevail	1372 (55.9%)	1082 (44.1%)	
		Control	622 (41.0%)	894 (59.0%)	
	Male				
		Prevail	311 (56.8%)	237 (43.2%)	
		Control	195 (43.6%)	252 (56.4%)	< 0.001
Mental health absences					
	Female				
		Prevail	599 (53.1%)	530 (46.9%)	
		Control	66 (19.9%)	265 (80.1%)	
	Male				
		Prevail	102 (87.9%)	14 (12.1%)	
		Control	80 (64.0%)	45 (36.0%)	< 0.001

## Discussion

In order to evaluate the Prevail intervention we assessed, (1) whether the people who underwent Prevail thought that the intervention achieved its aims, (2) whether Prevail produced changes in people’s mental health literacy (including stigma reduction), and (3) whether Prevail changed their ability to work (reduced absenteeism) as indicated by the number of days taken as sickness absence. We found strong evidence that Prevail achieved all three aims.

### Evaluation of the prevail intervention

Prevail Staff Intervention was administered to staff via a one-day group intervention. Most participants evaluated the duration and pace of the intervention as appropriate and that they were able to understand the Prevail content. Crucially, they also thought that they would be able to improve their own mental health, and be able to help other people with their mental health. Hence, Prevail has strong face value in its objectives and was found to be palatable and valuable to the workforce. This is important as the perceived value of any intervention is going to be an important factor as to whether people physically attend, and psychologically engage, with the intervention [23]. Clearly without this active participation and perceived value of the intervention there is little chance that it can affect attitudes and future behaviours [24]. A similar set of results was found for the Prevail Managers Intervention with similar implications.

### Changes in mental health literacy

The Prevail Intervention led to clear reductions in levels of self-stigma and anticipated stigma. These two scales are those most strongly associated with mental health [20]. There were also reductions in social distance and (lack of) disclosure/help-seeking for those in the active arm of the study. However, there were also reductions for those in the control arm, which we speculate may have been due to “leakage” of the Prevail programme to other members of the workforce that did not actively undertake the Prevail Intervention. While such leakage may be a problem for the evaluation of the Prevail Intervention as implemented by a RCT (see Limitation section), it can also be seen as a desirable consequence for the organisation and workforce as any improvement in mental health and reduction in stigma must be seen as a beneficial outcome.

### Changes in sickness absence

Producing an intervention that is valued by the workforce and which is able to change people’s attitudes and intentions to seek help is a good achievement. However, actually being able to change people’s future behaviour is a far more difficult task [25]. Importantly, the results of the RCT show strong support for the effectiveness of Prevail in reducing levels of sickness absence. In all analyses, Prevail reduced sickness absence (as indexed by the number of sick days taken) from the pre-intervention period to the post-intervention period for those people in the active arm of the study. This reduction in sickness absence was not found for staff members in the control group, who had not had the benefit of the Prevail intervention.

While the results of the Prevail intervention appear robust there are two surprising elements to the results that we did not expect: (1) the levels of sickness absence at the pre-intervention period were lower for the control group compared to the active group; and (2) levels of sickness absence increased for those in the control group between the pre- and post- intervention periods. We address each of these points in turn.

One of the purposes of conducting a RCT is to ensure that the effects of extraneous factors (such as levels of sickness absence pre-intervention) is equated across the two groups (active vs. control) - [26]. Clearly, this is not the case for our study. Examination of the randomisation process could identify no reason why the groups appeared to differ in baseline levels of sickness absence as the age, gender, and directorate of work was approximately equal across the active and control arms of the study. Thus, it appears that this difference is merely an unfortunate chance occurrence.

While we predicted reductions in sickness absence for those in the active arm of the study, it was expected that those in the control arm would have no change in the levels of sickness absence from pre- to post-intervention (as essentially nothing had happened with the control group and it was work practice as usual). However, levels of sickness absence actually increased. Hence, it would appear that there were factors at play that caused increased levels of sickness absence in the post-intervention period (Dec 2019-Feb 2020) compared to the pre-intervention period (Dec 2018-Feb 2019). While there may be other factors (e.g., increased rates of flu or other seasonal disorders) that were different between these time periods, the most salient factor is the onset of the COVID-19 pandemic. Although the first recorded case in Wales was at the end of our data collection period, in late February 2020, concerns about the on-coming pandemic and the impact of this internationally were evident much earlier as the disease spread across the world. This may have impacted anxiety levels and decisions to try and avoid busy workplaces at such a worrying time.

While the causes of the increase in rates of sickness absence in the control arm of the study are, at best, speculation, these unexpected increases in sick leave show the value of the RCT and of collecting pre-and post-intervention data to help with the interpretation of effects. If it is indeed the case that rates of sickness absence were higher in the data collection period post-intervention for the workforce as a whole (mimicking the effects in the control group), then the ***reduction*** in sickness absence seen for those members of staff who have had the benefit of the Prevail intervention is even more impressive.

#### Limitations to the RCT

The main limitation to the study were the unexpected differences in baseline levels of sickness absence between the two arms of the study (see discussion above). The study was also curtailed in some of its aims due to the onset of restrictions due to the COVID-19 pandemic that severely altered work practices and limited access of the researchers to the employees of the DVLA. Finally, the data on sickness absences could only be provided to the research team at the level of “groups” rather than individuals due to Data Protection issues. Hence, we were unable to examine the relationship between mental health literacy and stigma (as measured by the SASS) and sickness absence, or to data at the individual level. Future studies may benefit from research designs that are able to tie sickness rates to specific demographics such as history of mental health problems, gender, ethnicity, time in current employment, etc.

The study was also unable to prevent any “leakage” and contamination of the Prevail programme from people in the active arm of the study to those in the control arm despite efforts to do so via using cluster randomisation. However, it should be emphasised that any such leakage would have served to reduce the differences between the active and control arms the study and thus be conservative to our hypotheses.

Our original protocol for the study [7] included plans for a longer term follow-up of the effects of Prevail. Unfortunately, this part of the study was prevented due to the onset of the Covid-19 pandemic which caused massive changes in the workplace and prevented our access to the participants at the follow-up stage. Hence, the study was only able to demonstrate changes in attitudes and stigma over a short period of time. Further studies are needed to see if such changes are maintained over longer time periods.

Finally, while our data are supportive of the efficacy of Prevail they are not able to distinguish which parts of the Prevail intervention are effective (and which may not be). Indeed, part of the effect of Prevail may be accounted for by “placebo effects” in that merely taking part in any intervention that brings mental health and mental wellbeing to the forefront of peoples’ attention may be beneficial to mental wellbeing and therefore have effects on attitudes to mental health and in reduced sickness absences. Indeed, participants taking the Prevail intervention commented that the open acknowledgement of mental health challenges and stressors in the workplace led to feelings of validation and value by the employer.

## Conclusions

The Prevail programme is a novel workplace-based programme that is based on public health principles of trying to increase mental wellbeing and help-seeking in the whole workforce in the hope of reducing mental health problems and increasing help-seeking. As well as the obviously desirable effect for the individual and for healthcare services, these improvements mean a reduction in absenteeism and reduced workforce losses for the employer. In the present study we did not make any calculation of the cost effectiveness and economic impact of Prevail, and this is an area that requires further study in various settings and workplaces.

The Prevail programme is an important development in mental health interventions for the workplace. It was successful in its aims of producing a group intervention that was palatable to the workforce, reduced levels of stigma related to mental health problems, and reduced levels of sickness absence. While the full programme of research (see [7]) could not be completed due to the onset of the COVID-19 pandemic, the study was still well-powered and used a randomised control trial to evaluate the impact of the intervention. The Prevail Staff Intervention can be administered in just one-day (with an additional day for the Prevail Managers Intervention) and can be implemented in-house via a train-the-trainer programme. The delivery of the Prevail intervention by in-house trainers allows the intervention to be targeted to the specific needs of the workforce and to the culture of the employment setting, as this is familiar and understood by the trainers who form part of that culture and are accepted as such by the staff team. As such, we believe it is an effective and cost-sensitive intervention that would be valued by employees and could be used in many workforces and other settings (e.g., university and college students, the social care workforce, etc.) to improve rates of mental health literacy, reduce stigma related to mental health problems, improve levels of disclosure and help-seeking, and help employers to reduce sickness absence from work.

## Data Availability

The datasets generated during this research and/or analysed following completion of the current study are stored in a publicly available repository (Mendeley). Snowden, Robert (2022), “Reducing stigma and increasing workplace productivity due to mental health difficulties: A randomised control treatment trial (RCT) of a low intensity psychological intervention and stigma reduction programme for common mental disorder (Prevail).”, Mendeley Data, V1, doi: 10.17632/4ph3dr3vg5.1 The data shared are: (1) Results from the survey of opinions about the Prevail course. Data are at a group level. (2) an anonymised SPSS database that contains the item by item scores from the psychometric measures as well as the scale scores and demographic information, (3) an excel database containing data on number of sick days taken. These are at a group level only. The data was published at the time of provisional acceptance of this paper and will remain available indefinitely. Access will be open to anyone via the usual access to Mendeley.
